# Changes in Intratumor Blood Flow After Carbon-Ion Radiation Therapy for Early-Stage Breast Cancer

**DOI:** 10.1016/j.ijpt.2024.100018

**Published:** 2024-04-24

**Authors:** Kenta Ohmatsu, Tokuhiko Omatsu, Noriyuki Okonogi, Yoko Ikoma, Kazutoshi Murata, Riwa Kishimoto, Takayuki Obata, Shigeru Yamada, Kumiko Karasawa

**Affiliations:** 1 Department of Radiation Oncology, Tokyo Women’s Medical University School of Medicine, Tokyo, Japan; 2 QST Hospital, National Institutes for Quantum Science and Technology, Chiba, Japan; 3 Department of Radiation Oncology, Juntendo University Graduate School of Medicine, Tokyo, Japan; 4 Institute for Quantum Medical Science, National Institutes for Quantum Science and Technology, Chiba, Japan

**Keywords:** Dynamic contrast-enhanced, Magnetic resonance imaging, Carbon-ion radiation therapy, Breast cancer, Quantitative comparisons

## Abstract

**Purpose:**

This study aimed to quantify the changes in intratumoral blood flow after carbon-ion radiation therapy (CIRT) for early-stage breast cancer and analyze their clinical significance.

**Patients and Methods:**

We included 38 patients with early-stage breast cancer who underwent CIRT. Dynamic imaging was performed using a 3T superconducting magnetic resonance scanner to quantify the washin index (idx), which reflects contrast uptake, and washout idx, which reflects the rate of contrast washout from tumor tissue. The changes in the apparent diffusion coefficient, washin idx, and washout idx were examined before CIRT and at 1 and 3 months after treatment. Clinical factors and imaging features were examined using univariate and receiver operating characteristic curve analyses to identify factors predicting clinical complete response (cCR).

**Results:**

The median observation period after CIRT was 51 (range: 12-122) months. During the observation period, 31 of the 38 patients achieved cCR, and 22 achieved cCR within 12 months. Tumor size (*P* < .001), washin idx (*P* = .043), and washout idx (*P* < .001) decreased significantly 1-month after CIRT. In contrast, the apparent diffusion coefficient values (*P* < .001) increased significantly 1-month after CIRT. Univariate analysis suggested that the washin idx after 1 and 3 months of CIRT was associated with cCR by 12 months post-CIRT (*P* = .028 and .021, respectively). No other parameters were associated with cCR by 12 months post-CIRT. Furthermore, receiver operating characteristic curve analyses showed that the area under the curve values of washin idx after 1 and 3 months of CIRT was 0.78 (specificity 75%, sensitivity 80%) and 0.73 (specificity 75%, sensitivity 71%), respectively.

**Conclusion:**

Tumor changes can be quantified early after CIRT using contrast-enhanced magnetic resonance imaging in patients with breast cancer. Washin idx values 1 and 3 months after CIRT were associated with cCR within 12 months post-CIRT.

## Introduction

The most frequent malignancy in women is breast cancer. In 2020, 2.3 million new cases of breast cancer were diagnosed globally, representing more than 10% of all newly diagnosed cancers.^1^ Breast-conserving surgery is currently the standard treatment for early-stage breast cancer, followed by systemic therapy and postoperative radiation therapy (RT). In recent years, the number of older adult patients with breast cancer in Japan has increased,^2^ along with the proportion of patients who are unwilling or unable to undergo surgery because of its complications. Thus, there is a growing demand for nonsurgical therapy in patients with early-stage breast cancer. Nonsurgical treatment modalities, such as radiofrequency ablation, focused ultrasound, and cryotherapy, have been reported as potential therapeutic options for managing early-stage breast cancer. Radiofrequency ablation has gained recognition and is currently being integrated into clinical practice.^3^ However, these treatments require anesthesia or are accompanied by pain.

Proton beam therapy and carbon-ion RT (CIRT) are charged-particle therapies with excellent dose distributions. Numerous clinical studies using CIRT for different types of cancers have yielded positive results, owing to the improved dosage distribution and biological benefits of their increased linear energy transfer.^4^, ^5^ We have applied CIRT as a nonsurgical therapy for treating early-stage breast cancer.^6^, ^7^, ^8^ Treating early-stage breast cancer via CIRT does not require sedation or anesthesia; moreover, no pain is associated with irradiation; therefore, it is a noninvasive treatment comparable with other nonsurgical treatments for early-stage breast cancer. Furthermore, treating early-stage breast cancer with CIRT has shown favorable results.^9^, ^10^

Medical imaging plays a crucial role in the nonsurgical management of breast cancer. Magnetic resonance (MR) imaging (MRI) of the breast provides diverse functional information using diffusion-weighted images (DWI) and contrast agents. Recent studies have shown that MRI techniques can help distinguish benign from malignant tumors and identify specific breast cancer subtypes.^11^, ^12^ Furthermore, the analysis of suitable MR images holds value in the prediction of the initial effectiveness of preoperative systemic therapy or the postoperative prognosis for patients with breast cancer.^13^, ^14^, ^15^, ^16^ However, no study has reported pre and post-CIRT MRI changes in patients with early-stage breast cancer.

The purpose of this study was to quantify the functional changes in early breast cancer after applying CIRT using MR images. Furthermore, we aimed to investigate the relationship between these parameters and tumor reduction.

## Materials and methods

### Study design

This single-center retrospective study included patients with early-stage breast cancer who underwent CIRT at our institution between April 2013 and May 2022. This study was approved by our institutional review board and ethical committee (approval number: N21-019). The study's retrospective design allowed for the waiver of the written informed consent requirement. Patients and their families had the option to decline participation in the study, and the details of the opt-out policy were placed on the institution's website.

### Study cohort

The study included patients enrolled and treated in clinical trials of CIRT for early-stage breast cancer at our institution during the study period.^6^, ^7^, ^8^ All patients received CIRT in single or 4 fractions, followed by systemic therapy based on their breast cancer subtypes. In the Breast II protocol,^7^ patients received whole breast irradiation 50 Gy/25 fractions or 42.5 Gy to 43.2 Gy/16 fractions started after a 1-month follow-up to ensure there were no adverse events, such as skin damage post-CIRT. The MR images were obtained before and up to 3 months after CIRT according to clinical trial protocols and thereafter MRI was performed every 3 months until clinical complete response (cCR) was confirmed.^6^, ^7^, ^8^ The patients who had undergone a planned tumor resection, had local recurrence, or had not undergone dynamic MRI were excluded. Patients with subtypes other than the luminal type, such as triple-negative and human epidermal growth factor type 2 types, were excluded because systemic therapy could affect the analysis.

### MR image acquisition

All MR scans were acquired using 3T superconducting equipment (Skyra, Siemens, Germany) with a 4-channel breast coil. The contrast agent was administered using an automatic injector (Sonic Shot GX; Nemoto Kyorindo). The DWIs were obtained using a multishot echo-planar imaging sequence (Resolve) with b-values of 0 and 1000 s/mm^2^. Dynamic MRI was performed using 3D gradient echo T1-weighted imaging (t1_fl3d) in the axial plane. Gadolinium contrast agents (Gadovist, Magnescope, Guerbet) were injected at a rate of 0.1 mmol/kg at 2 mL/second, followed by 60 mL of saline injection. Ten dynamic MRI scans were performed after acquiring 2 T1-weighted images with different flip angles for calculating the T1 map. At 252 seconds after the initiation of the contrast agent injection, fat-suppressed t1_fl3d was performed in the axial plane. The imaging parameters and dynamic MRI protocols are listed in Table 1.

**Table 1:** MRI imaging parameters.

	FOV	Slice thickness	TR	TE	FA	Matrix	Scanning time
	(mm)	(mm)	(ms)	(ms)	(degree)		(min: sec)
DWI	340	4	10,700	83, 134	180	214 x 214	4: 50
T1map	360	2	4.92	2.46	3, 15	307 x 384	1: 24
Dynamic	360	2	4.5	2.46	12	307 x 384	0: 42
Fat suppressed Gd	340	0.8	8.52	4.92	15	363 x 488	3: 17

**Abbreviations:** DWI, diffusion-weighted image; FA, flip angle; FOV, field of view; Gd, gadolinium; TR, repetition time; TE, echo time.

### Image analysis

The Breast Imaging Reporting and Data System MR guidelines outline the categorization of the time-intensity curves derived from dynamic MRI for evaluating blood flow.^17^ The time-intensity curve pattern analysis is helpful for qualitative diagnosis, including the differentiation between benign and malignant breast tumors.^18^ However, comparing patients pre and post-treatment using qualitative evaluation is difficult. As the contrast agent concentration and the relaxation rate (R1 [1/T1]) value are proportional, it is possible to convert the MRI signal values into contrast agent concentrations. By converting MRI signals into contrast agent concentrations, the attention of the contrast agent in the tissue can be estimated using a compartment model.

Ktrans indicates the transfer constant of the contrast agent between the plasma and extravascular extracellular space, reflecting vascular permeability and blood flow within the tissue. Kep represents the rate constant at which the contrast agent returns from the extravascular extracellular space to plasma, reflecting the washout of the contrast agent from the tissue. According to the compartment model, contrast agent concentration Ct(t) is represented by the convolution integral of the arterial input function Cp(t) with parts Ktrans and Kep. Ktrans determines the amplitude of the time-concentration curve (TCC), and Kep determines its shape.^19^, ^20^ The acquisition time of one phase of this dynamic MRI was 42 seconds, and it was difficult to obtain the arterial input function because of the low time resolution.

We defined the area under the TCC from the start of the contrast agent injection up to 2 minutes during early contrast enhancement as the washin index (idx). The ratio of the integral values of the TCC was termed the washout idx. It is postulated that the washin idx reflects the Ktrans value and the washout idx represents the Kep value.^21^ The specific washin idx and washout idx estimation methods are presented in Supplementary Data 1. Therefore, indices that do not require arterial input functions enable comparisons between different cases and pre and post-treatments, which are unachievable through qualitative evaluations.^22^, ^23^, ^24^

Using the dynamic MRI analysis software developed in MATLAB (MathWorks: R2022b; https://www.nirs.qst.go.jp/rd/structure/rccpt/amr-diag/download-apps/), we created washin idx and washout idx maps from the dynamic MR images. From the DWIs, apparent diffusion coefficient (ADC) maps were generated using the MR images. The ADC, washin idx, and washout idx maps, and post-contrast fat-suppressed 3D T1-weighted images were transferred to a workstation (MIM Maestro, MIM Software inc). Using the contour extraction feature of MIM Maestro, tumor contours were semiautomatically extracted from postcontrast fat-suppressed 3D T1-weighted images. The tumor contours were determined by a consensus between a diagnostic radiologist and a radiation oncologist, and the tumor volume was calculated. To ensure that the volume of interest (VOI) did not include normal mammary glands or the surrounding adipose tissue, the VOI was set to cover 80% of the tumor volume. The ADC, washin idx, and washout idx maps were fused with postcontrast fat-suppressed 3D T1-weighted images, and the VOI was copied to determine the median ADC, washin idx, and washout idx values for the entire tumor.

### Determination of the effectiveness of the treatment

The treatment efficacy was evaluated using the Response Evaluation Criteria in Solid Tumors guidelines version 1.1,^25^ Furthermore, the MR images were used for all patients. The absence of an enhancement at the treatment site was defined as a cCR.

### Statistical analysis

The changes in ADC, washin idx, and washout idx before, and 1 and 3 months after treatment were examined using repeated measures analysis of variance; *P*-values were corrected using the Bonferroni correction. The clinical factors (age, pretreatment tumor size, irradiation fractions, Tumor, Node, Metastasis stage, Ki-67 index) and imaging features were examined for factors predicting cCR using univariate analysis. Continuous variables were analyzed using the Mann-Whitney *U* test, and nominal variables were analyzed using Fisher's exact test. Using receiver operating characteristic (ROC) curve analysis, the area under the curve was calculated, and factors predicting cCR were analyzed. Furthermore, univariate analysis (Mann-Whitney *U* test) was performed for each irradiation frequency to investigate the effect of each imaging factor on the changes over time. All statistical procedures were performed using R language (R Development Core Team; version 4.3.1; The R Foundation for Statistical Computing); moreover, *P* < .05 was considered statistically significant.

## Results

Out of the 57 enrolled patients, 38 were included in the analysis. Supplementary data 2 depicts a flowchart of the patient selection procedure. The median patient age was 62 years, ranging from 37 to 91 years. The observation period after CIRT ranged from 12 to 122 months, with a median of 51 months. Thirty-five patients had invasive ductal carcinoma and 3 had ductal carcinoma in situ. The CIRT doses were 46 GyRBE/1 fraction, 50 GyRBE/1 fraction, 52.8 GyRBE/4 fractions, and 60 GyRBE/4 fractions in 2, 4, 5, and 27 patients, respectively. The patient and tumor characteristics are shown in Table 2. Twelve patients received whole breast irradiation after CIRT. MRI was performed for all patients before CIRT, and for 23 and 33 patients 1 and 3 months after CIRT, respectively.

**Table 2:** Patient and tumor characteristics.

Factor	Median/group	Range, *n*
Age	62	37-91
Tumor size (mm)	13	6-20
Pathological Diagnosis	IDC	35
	DCIS	3
TNM staging	0 (TisN0M0)	3
	Ⅰ (T1N0M0)	35
Subtype	Luminal type	38
Location	Left breast	12
	Right breast	26
Dose (GyRBE/fractions)	52.8/4	5
	60/4	27
	46/1	2
	50/1	4

**Abbreviations:** DCIS, ductal carcinoma in situ; IDC, invasive ductal carcinoma; RBE, relative biological effectiveness; TNM, Tumor, Node, Metastasis.

**Figure 1:**
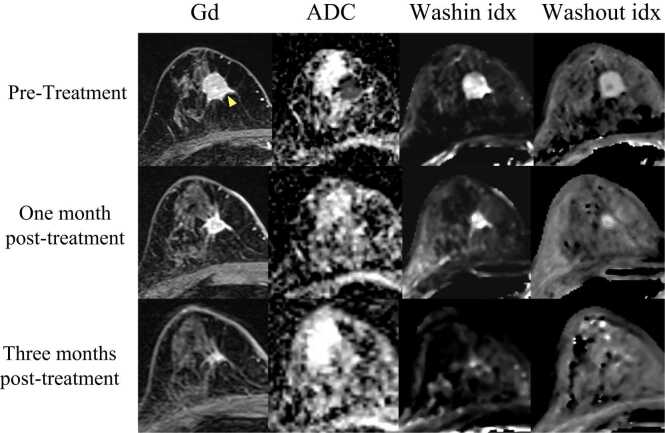
Examples of changes in ADC and blood flow after carbon-ion beam therapy. A 72-year-old woman underwent carbon-ion radiotherapy 46 GyRBE/1 fraction for a 16 mm breast cancer (invasive ductal carcinoma) in the right A region. Her pretreatment ADC was 0.89 x 10^-3^ mm^2^/s, washin idx was 305 x 10^-3^ mM/min, and washout idx was 223 x 10^-3^/min. At 1 month and 3 months after starting the treatment, ADC was 1.65 x 10^-3^ mm^2^/s, 1.63 x 10^-3^ mm^2^/s, washin idx was 263 x 10^-3^ mM/min, 52 x 10^-3^ mM/min, washout idx was 198 x 10^-3^/min, 183 x 10^-3^/min. Abbreviations: ADC, apparent diffusion coefficient; Gd, gadolinium.

**Figure 2:**
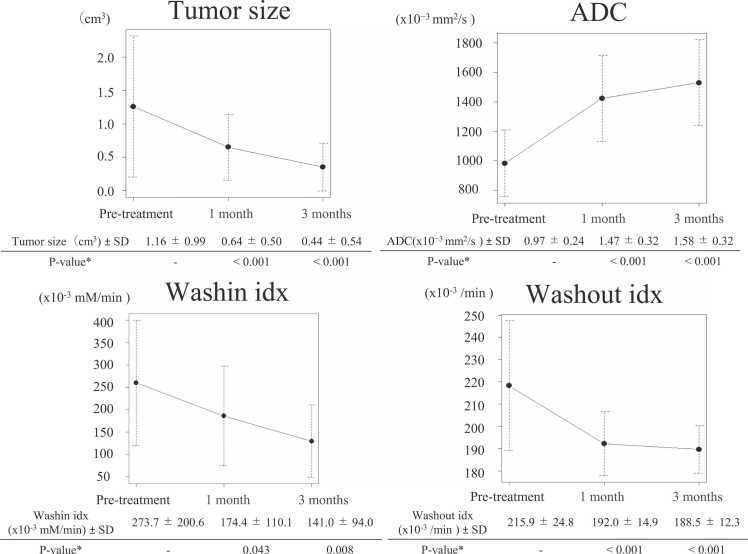
The changes in tumor volume, ADC, washin idx, and washout idx before CIRT and 1 and 3 months after CIRT. At 1-month and 3 months after the start of treatment, the ADC significantly increased, and tumor size, washin idx, and washout idx considerably decreased compared with their values before treatment. *P-values were compared with the pretreatment values and adjusted using the Bonferroni method. Abbreviation: ADC, apparent diffusion coefficient.

Washin idx, washout idx, and ADC levels were quantified in all 38 patients. Figure 1 shows the changes in each evaluation method for a representative case over time. Figure 2 shows the changes in tumor volume, ADC, washin idx, and washout idx before CIRT and 1 and 3 months after CIRT. Supplementary Data 3 shows the changes in each factor for all 38 patients. The average value ± standard deviation (SD) of tumor volume, ADC, washin idx, and washout idx before CIRT were 1.16 ± 0.99 cm^3^, 0.97 ± 0.24 x 10^-3^ mm^2^/second, 273.7 ± 200.6 x 10^-3^ mM/minute, and 215.9 ± 24.8 x 10^-3^/minute, respectively. The tumor size (*P* < .001), washin idx (*P* = .043), and washout idx (*P* < .001) decreased significantly 1-month after CIRT. In contrast, the ADC values (*P* < .001) increased significantly 1-month after CIRT. When classified by CIRT fractions (single fraction vs 4 fractions), there were no statistical differences in any of the parameters analyzed (Supplementary Data 4).

**Figure 3:**
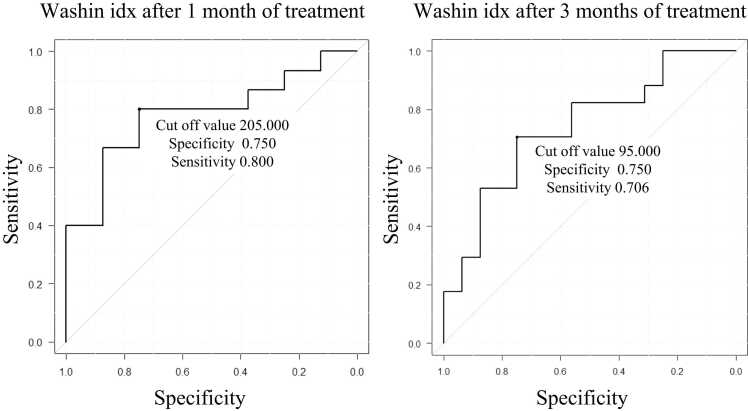
ROC curves of the washin index values after 1 and 3 months. Abbreviation: ROC, receiver operating characteristic.

Subsequently, we analyzed the association between each parameter and cases that led to cCR. The median time to achieve cCR was 12 months. During the observation period, 31 of the 38 patients achieved cCR; furthermore, 22 patients achieved cCR within 12 months. Tumor enhancement in patients who did not achieve cCR within 12 months also gradually shrank over time and eventually disappeared. Table 3 shows the results of the univariate and ROC analyses of the factors that predicted cCR within 12 months of treatment. Univariate analysis suggested that washin idx values after 1 and 3 months of CIRT were associated with cCR at 12 months post-CIRT (*P* = .028 and .021, respectively). Figure 3 shows the ROC curves of the washin idx values after 1 and 3 months. No other parameters were associated with cCR at 12 months post-CIRT. The ROC curve analyses showed that the area under the curve values of washin idx after 1 and 3 months of CIRT was 0.78 (specificity 75%, sensitivity 80%) and 0.73 (specificity 75%, sensitivity 71%), respectively. No clinical factors (age, pretreatment tumor size, irradiation fractions, Tumor, Node, Metastasis Stage, Ki-67) were identified as predictive indicators of cCR at 12 months post-CIRT.

**Table 3:** Univariate and ROC curve analysis of factors predicting cCR within 12 months after treatment.

Factor	Patients without cCR in 12 months (*n* = 16) (Median (IQR))	Patients with cCR in 12 months (*n* = 22) (Median (IQR))	*P*-value	AUC	Cut off value (specificity, sensitivity)
Age	59 (50-66)	64 (52-69)	.459	0.43	56 (0.62, 0.36)
Pretreatment tumor size (mm)	11.5 (10.7-17)	14 (10.5-16.7)	.858	0.48	16 (0.31, 0.72)
Irradiation fractions (1/4)	*n* = 5/11	*n* = 1/21	.064	-	-
TNM Stage (0/Ⅰ)	*n* = 1/15	*n* = 2/20	1.000	-	-
Ki-67 (%)	10 (7.7-36)	15 (8.4-19)	.835	0.47	19 (0.33, 0.76)
Washin idx (x10^-3^ mM/min)					
Pretreatment	215 (191-293)	224 (121-318)	.848	0.52	164 (0.94, 0.36)
1-month post-treatment	236 (211-262)	100 (73-202)	**.028**^b^	**0.78**^b^	**205 (0.75, 0.80)**^b^
3 months post-treatment	159 (94-214)	85 (66-133)	**.021**^b^	**0.73**^b^	**95 (0.75, 0.71)**^b^
1-month post-treatment reduction rate^a^	0.066 (−0.09 to 0.11)	0.38 (−0.005 to 0.62)	.190	0.67	0.29 (0.88, 0.67)
3 months post-treatment reduction rate^a^	0.31 (0.052-0.66)	0.65 (0.26-0.83)	.157	0.65	0.44 (0.63, 0.65)
Washout idx (x10^-3^/min)					
Pretreatment	215 (209-237)	215 (198-223)	.209	0.62	232 (0.38, 0.91)
1-month post-treatment	204 (194-208)	186 (181-196)	.121	0.70	198 (0.75, 0.80)
3 months post-treatment	189 (184-215)	184 (183-213)	.101	0.66	187 (0.56, 0.76)
1-month post-treatment reduction rate^a^	0.15 (0.058-0.17)	0.084 (0.055-0.14)	.506	0.40	0.076 (0.38, 0.60)
3 months post-treatment reduction rate^a^	0.13 (0.091-0.18)	0.12 (0.088-0.17)	.763	0.46	0.10 (0.37, 0.71)
ADC (x10^−3^ mm^2^/s)					
Pretreatment	953 (886-1070)	876 (814-1055)	.487	0.43	1013 (0.63, 0.36)
1-month post-treatment	1352 (1268-1507)	1521 (1296-1688)	.466	0.60	1371 (0.63, 0.73)
3 months post-treatment	1424 (1342-1600)	1680 (1528-1781)	.053	0.69	1596 (0.75, 0.64)
1-month post-treatment increase rate^a^	0.56 (0.46-0.63)	0.50 (0.25-0.92)	1.000	0.50	0.85 (1.00, 0.33)
3 months post-treatment increase rate^a^	0.54 (0.38-0.67)	0.57 (0.29-1.15)	.276	0.61	0.57 (0.68, 0.59)
Tumor volume (cm^3^)					
Pretreatment	0.64 (0.37-2.31)	0.95 (0.51-1.11)	.953	0.51	1.12 (0.43, 0.77)
1-month post-treatment	0.69 (0.31-1.27)	0.43 (0.28-0.68)	.401	0.61	0.89 (0.50, 0.86)
3 months post-treatment	0.28 (0.15-0.76)	0.31 (0.11-0.49)	.482	0.57	0.31 (0.50, 0.59)
1-month post-treatment reduction rate^a^	0.37 (0.28-0.46)	0.45 (0.33-0.63)	.357	0.63	0.45 (0.75, 0.53)
3 months post-treatment reduction rate^a^	0.53 (0.42-0.66)	0.64 (0.45-0.79)	.160	0.64	0.63 (0.68, 0.58)

**Abbreviations:** ADC, apparent diffusion coefficient; AUC, area under the curve; cCR, clinical complete response; IQR, interquartile range; TNM, Tumor, Node, Metastasis.

a Rates of change for each factor were compared to pretreatment.

b Bolded items indicate that the Area under the curve became larger with significant differences with p-values < 0.05.

## Discussion

To the best of our knowledge, this is the first report to quantify MR changes after CIRT for early-stage breast cancer. The use of washin idx and washout idx allowed for quantitative, rather than qualitative, comparisons over time between different cases, along with comparisons before and after treatment in the same case. Furthermore, quantification was possible for all the lesions, on which it was attempted, regardless of the breast tumor size. Thus, our method may be applicable for quantifying the imaging characteristics of other tumors after RT.

The washin idx reflects the amount of contrast uptake in the early phase, while washout idx demonstrates the rate of contrast washout (Kep) from the tumor tissue.^19^, ^20^, ^21^ Furthermore, ADC is a measure of the magnitude of diffusion of water molecules within the tissues, reflecting the cell density within the tumor tissue.^26^ This study demonstrated that the tumor blood flow and density changed dramatically as early as 1-month after CIRT. As shown in Supplementary Data 3, although washin idx showed no changes, the ADC increased and washout idx decreased in many cases 1-month after CIRT, resulting in the *P*-values for the ADC and washout idx being much smaller than those for washin idx. Notably, RT damages not only the tumor cells but also the endothelial cells within the tumor.^27^ Moreover, vascular injury occurs 7 days after a single CIRT irradiation.^28^ Therefore, washout idx, which reflects the rate of contrast washout from the tumor, may be highly reduced early and may reflect decreased tumor blood flow owing to the intratumor circulation dysfunction. The ADC increase may reflect an increase in the extravascular space owing to changes in vascular conditions. The reason for the relatively small decrease in washin idx expression remains unclear. The washin idx reflects both Kep and extracellular space fraction.^19^ Therefore, the off-setting effects of reduced Kep and increased extracellular space may have resulted in a weak change in washin idx. Further studies are needed to clarify whether this trend is specific to CIRT or conventional RT.

This study showed that washin idx values at 1 and 3 months after CIRT were associated with the occurrence of a cCR within 12 months after CIRT. Predicting the efficacy of radiation therapy is often challenging; therefore, it is important to identify a single, highly sensitive, and specific index. Surrogate markers that predict the occurrence of cCR in nonsurgical therapy are clinically important for appropriately determining whether post-treatment is required. Recent studies on preoperative chemotherapy for breast cancer have shown that both ADC and tumor blood flow predictor the occurrence of pathological complete response to the same extent.^29^, ^30^, ^31^, ^32^, ^33^ Preoperative chemotherapy for breast cancer causes tumor necrosis and decreases tumor vascularity.^26^ Despite a relatively large ADC value, washin idx expression decreased in our study (Table 3). Therefore, it is difficult to conclude that the low washin idx was due to the shrinking of the extracellular space. Tumor blood flow and volume also contribute to contrast effects in the early phases of dynamic contrast-enhanced MRI.^18^ Since no significant difference was observed, that depended on the treatment effect in washout idx, it is possible that the decrease in tumor blood volume is greater in patients with good treatment effects than in patients with poor treatment effects. It may be necessary to verify whether there are differences in the effects of CIRT and chemotherapy on ADC and tumor blood flow, both of which have cell-killing effects in vivo or in other ways. Overall, the above discussion suggests that the washout idx and ADC values are effective in confirming the reliability of the irradiation process, whereas the washin idx value is effective in predicting the treatment efficacy.

In recent years, considerable research has been conducted on various modalities for predicting treatment response in breast cancer. Residual tumor metabolic activity on ^18^F-fluorodeoxyglucose positron emission tomography indicates an active disease, and the loss of metabolic activity is predictive of a successful therapeutic response.^34^, ^35^ Furthermore, an early post-treatment metabolic complete response could be a more precise predictor of the overall survival than the late post-treatment metabolic complete response in patients undergoing ^18^F-fluorodeoxyglucose positron emission tomography during preoperative chemotherapy for breast cancer.^36^ Combining multiple diagnostic modalities may improve the predictive accuracy of cCR. Moreover, It may be possible to predict the efficacy of nonablative treatment for breast cancer or to quantitatively evaluate the MRI findings for breast cancer and other cancers. Further analysis with a large number of cases and cancer types is warranted.

No reports were found on preoperative partial breast irradiation (PBI) using proton therapy. However, preoperative PBI for low-risk breast cancer patients has been reported in the ABLATIVE trial from the Netherlands,^37^ SABR by Barry et al,^38^ and stereotactic body radiation therapy and intensity modulated radiation therapy by Shibamoto et al^39^ Additionally, registration has begun for phase 2 study ABLATIVE-2,^40^ which involves MRI-guided preoperative PBI targeting low-risk breast cancer patients. This trial also includes an assessment of treatment response using MR imaging. But currently, there are no reports on the quantitative examination of changes in dynamic MRI or ADC following radiation therapy.

This study had several limitations, including the fact that it was a single-center retrospective analysis with a small number of cases. First, the assessment of cCR has been solely conducted through contrast-enhanced MRI, without pathological examination via biopsy or surgery. There remains a possibility of residual microscopic tumor tissue even when tumor enhancement disappears on contrast-enhanced MRI. Moreover, following after CIRT, the disappearance of tumor enhancement occurred between 3 and 24 months, showing considerable variability in the duration of enhancement disappearance. Nevertheless, even when MRI indicated disappearance of enhancement, we continued with vigilant follow-up. If tumor recurrence was suspected based on factors like an increase in tumor size or washin idx and washout idx on contrast-enhanced MRI, a biopsy was conducted to diagnose tumor recurrence. In cases of confirmed recurrence, additional surgical intervention was implemented. This approach of continued monitoring supports the validity of determining cCR using contrast-enhanced MRI.

Second, in this study, the tumor sizes were a maximum of 2 cm, relatively small, and image fusion was employed to minimize measurement errors. However, distortions due to differences in MR imaging sequences, misalignment caused by sequence variations, and errors from the partial volume effect due to varying slice thicknesses may have amplified inaccuracies.

Third, in this study, the arterial input function, which was necessary to correct the contrast agent concentration in the tissue, was assumed to be a constant when determining the washin idx and washout idx. Further improvements in accuracy may be possible in the future by conducting analyses that consider the variable nature of the arterial input function.

Fourth, in this study, due to the limited number of cases in a single protocol, multiple protocols were analyzed together, resulting in a variety of treated doses and dose fractions. This variation in treatment regimens likely contributed to the significant variability observed in the changes of washin idx, washout idx, and ADC.

## Conclusion

In conclusion, we demonstrated that contrast-enhanced MRI was able to quantify the tumor changes early after CIRT for breast cancer. The washin idx values 1 and 3 months after CIRT were associated with cCR within 12 months post-CIRT. Our method may help quantify the imaging characteristics of other tumors after RT; however, further studies are needed to examine this.

## Ethics statement

All patient data has been collected under an internal review board (IRB) approved protocol.

## Funding

This study received no specific grants from government, commercial, or non-profit funding agencies.

## Author Contributions

**Kenta Ohmatsu**: Conceptualization, Data curation, Formal analysis, Investigation, Writing – original draft. **Tokuhiko Omatsu**: Conceptualization, Data curation, Formal analysis, Investigation, Writing – original draft. **Noriyuki Okonogi**: Writing – original draft. **Yoko Ikoma**: Methodology, Software, Writing – review and editing. **Kazutoshi Murata**: Writing – review and editing. **Riwa Kishimoto**: Writing – review and editing. **Takayuki Obata**: Methodology, Software, Writing – review and editing. **Shigeru Yamada**: Writing – review and editing. **Kumiko Karasawa**: Supervision, Writing – review and editing. All authors commented on the previous versions of the manuscript and have read and approved the final version.

## Declaration of Conflicts of Interest

The authors declare that they have no known competing financial interests or personal relationships that could have appeared to influence the work reported in this paper.
