# Supplementary material for: Changes in Intratumor Blood Flow After Carbon-Ion Radiation Therapy for Early-Stage Breast Cancer
Source: Int J Part Ther. 2024 Apr 24;12:100018. doi: 10.1016/j.ijpt.2024.100018 (PMC11252070; doi:10.1016/j.ijpt.2024.100018)
Supplement: Supplementary file 1 — Supplementary material [file mmc1.docx]

**Supplementary data 1**

Estimation of Washin idx and Washout idx

Based on the two-compartment model with the plasma and tissue compartments, the concentration of contrast agent in the tissue, *C*_t_(*t*), is expressed as follows [19];

$\left. C_{t}\left( t \right)=K_{trans}\cdot e^{-k_{ep}t}\otimes\boldsymbol{C}_{\boldsymbol{p}}(t \right)$ (1)

where *K*_trans_ is the transfer constant of the contrast agent from the plasma to the tissue, reflecting the vascular permeability and blood flow in the tissue, *k*_ep_ is the transfer rate constant from the tissue to the plasma, reflecting the Washout of contrast agent from the tissue, *C*_p_ is the concentration of contrast agent in the plasma, and ⊗ is the convolutional arithmetic operation. According to this equation, *K*_trans_ determines the amplitude of time-concentration curve and *k*_ep_ determines the shape of time-concentration curve depending on the Washout rate.

To evaluate these *K*_trans_ and *k*_ep_ values by the simple arithmetic operation, Washin idx, and Washout idx are introduced as follows [20, 21];

$$washin-idx=\int_{0}^{T_{early}} C_{t}\left( t \right)dt$$

(2)

$$washout-idx=\frac{\int_{0}^{T_{delay}} C_{t}\left( t \right)dt}{\int_{0}^{T_{delay}} {t\cdot C}_{t}\left( t \right)dt}$$

(3)

The Washin idx, that is the integration of *C*_t_ at the early phase, reﬂects the magnitude of inﬂux of the contrast agent, and is related to the *K*_trans_ value, as the Washout component that depends on *k*_ep_ is relatively small at the early phase. Meanwhile, the Washout idx is a function of only *k*_ep_, by considering Eq.1 and 3, based on the assumption that *C*_p_ is identical within a measured region of a subject.

In this study, the Washin idx value was estimated by the integration of *C*_t_ from 0 to 2 min (*T*_early_ = 2 min in Eq.2), and the Washout idx value was estimated by the integration of *C*_t_ from 0 to 10 min (*T*_delay_ = 10 min in Eq.3).
