# Supplementary material for: Changes in Intratumor Blood Flow After Carbon-Ion Radiation Therapy for Early-Stage Breast Cancer
Source: Int J Part Ther. 2024 Apr 24;12:100018. doi: 10.1016/j.ijpt.2024.100018 (PMC11252070; doi:10.1016/j.ijpt.2024.100018)
Supplement: Supplementary file 2 — Supplementary material [file mmc2.pdf]

Supplementary data 2 : Flow diagram of the patient selection process for this study

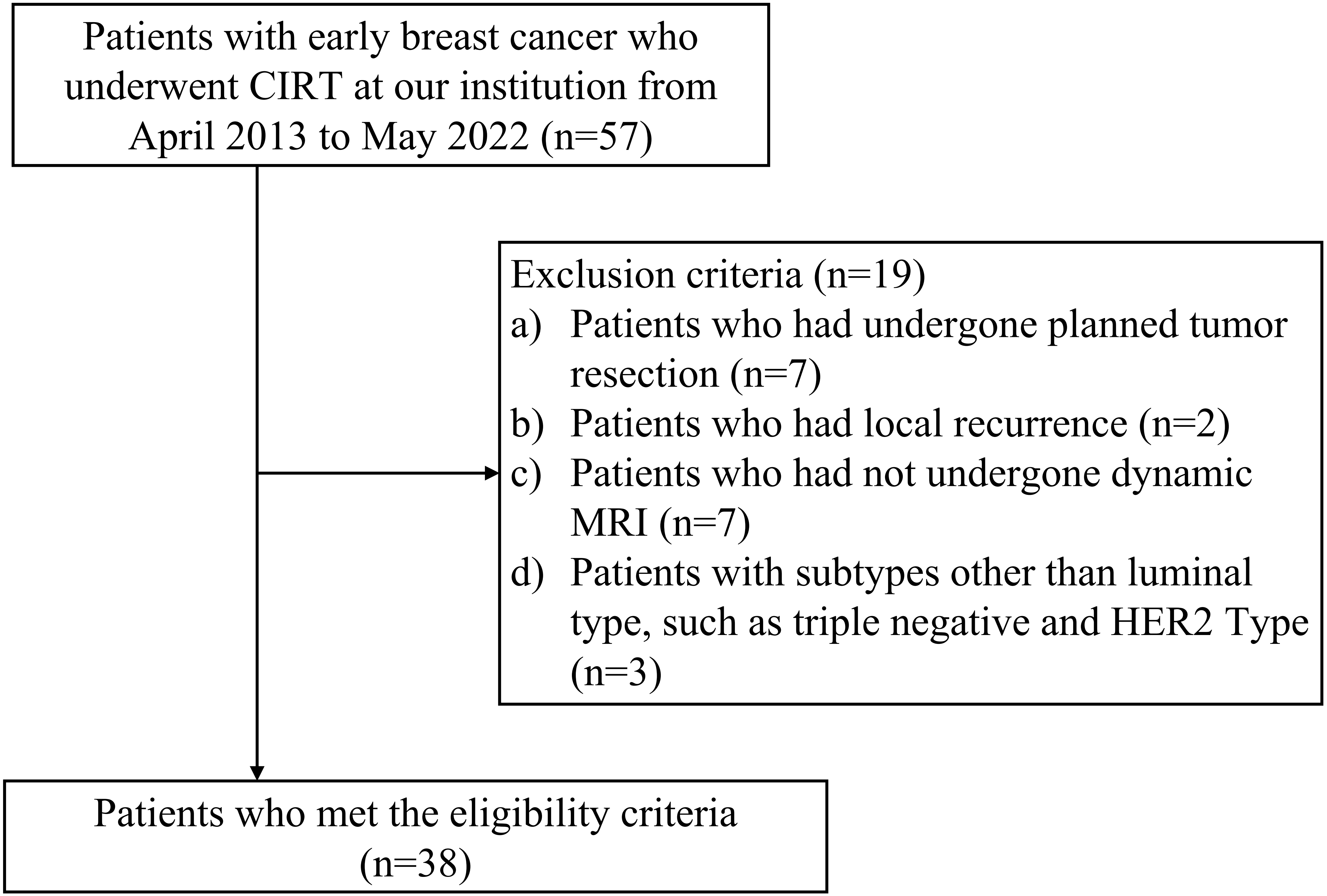

Abbreviations: CIRT= Carbon-ion Radiation Therapy MRI = Magnetic Resonance Imaging
HER2 = Human epidermal growth factor type 2
