# Supplementary material for: Changes in Intratumor Blood Flow After Carbon-Ion Radiation Therapy for Early-Stage Breast Cancer
Source: Int J Part Ther. 2024 Apr 24;12:100018. doi: 10.1016/j.ijpt.2024.100018 (PMC11252070; doi:10.1016/j.ijpt.2024.100018)

Supplementary data 3

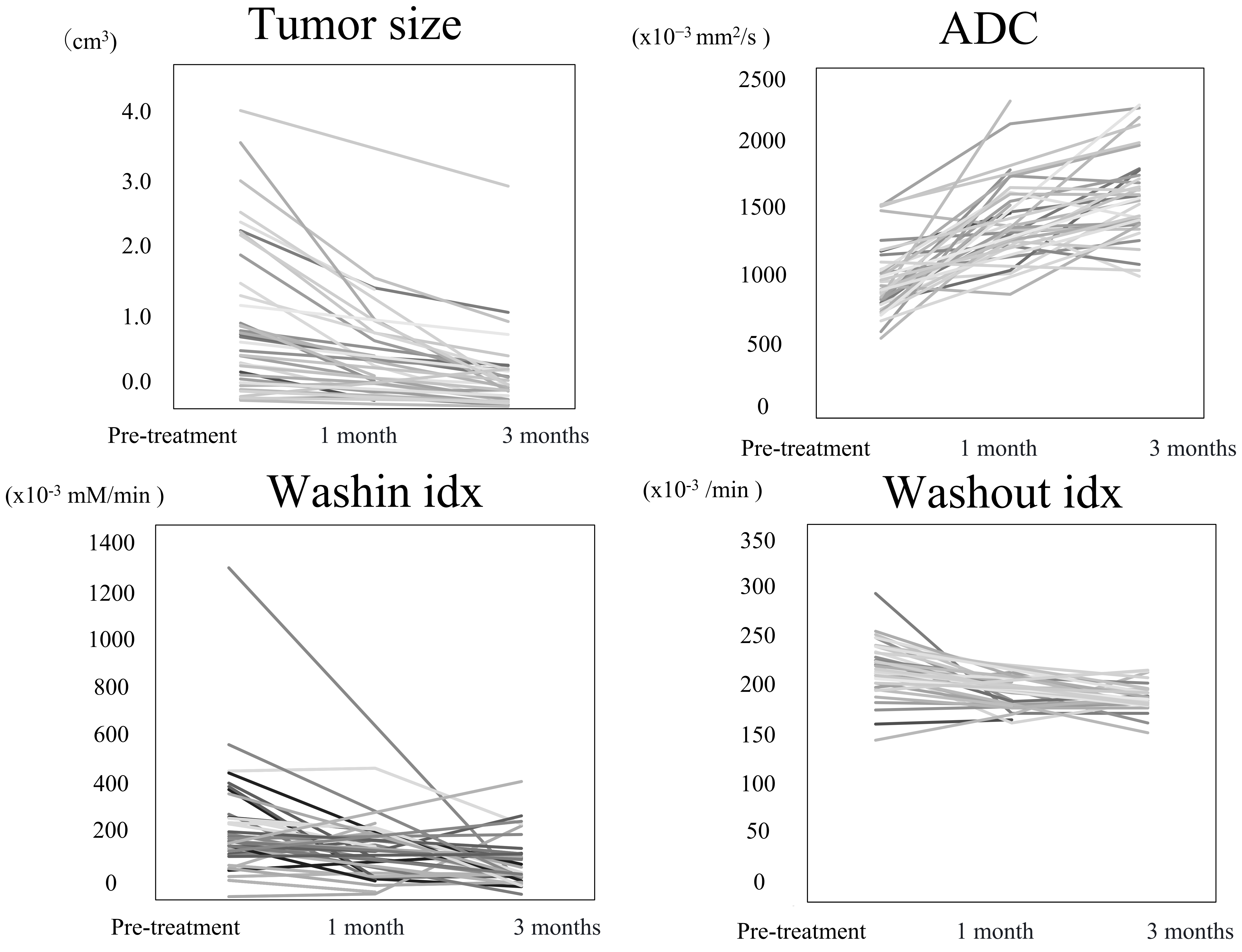

Supplement: Supplementary file 3 — Supplementary material [file mmc3.pdf]
