# Supplementary material for: Changes in Intratumor Blood Flow After Carbon-Ion Radiation Therapy for Early-Stage Breast Cancer
Source: Int J Part Ther. 2024 Apr 24;12:100018. doi: 10.1016/j.ijpt.2024.100018 (PMC11252070; doi:10.1016/j.ijpt.2024.100018)
Supplement: Supplementary file 4 — Supplementary material [file mmc4.docx]

Supplementary data 4. Univariate analysis of the rate of change of each image parameter grouped by the number of irradiations (1 vs. 4 fractions)

|  | 1 fraction (n=6) (Median (IQR)) | 4 fractions (n=32) (Median (IQR)) | p-value |
| --- | --- | --- | --- |
| Washin idx (x10^-3^ mM/min) reduction rate* |  |  |  |
| 1 month post-treatment | 0.13 (0.13–0.13) | 0.22 (-0.11–0.59) | 0.957 |
| 3 months post-treatment | 0.23 (-0.47–0.71) | 0.44 (0.25–0.75) | 0.508 |
|  |  |  |  |
| Washout idx (x10^-3^ /min) reduction rate* |  |  |  |
| 1 month post-treatment | 0.11 (0.11–0.11) | 0.09 (0.05–0.16) | 0.957 |
| 3 months post-treatment | 0.11 (0.056–0.17) | 0.13 (0.092–0.19) | 0.569 |
|  |  |  |  |
| ADC (x10^−3^ mm^2^/s) increase rate* |  |  |  |
| 1 month post-treatment | 0.85 (0.85–0.85) | 0.51 (0.29–0.76) | 0.435 |
| 3 months post-treatment | 0.50 (0.39–0.55) | 0.56 (0.36–0.98) | 0.348 |
|  |  |  |  |
| Tumor volume (cm^3^) reduction rate* |  |  |  |
| 1 month post-treatment | 0.49 (0.49–0.49) | 0.42 (0.31–0.59) | 0.783 |
| 3 months post-treatment | 0.39 (0.28–0.64) | 0.61 (0.45–0.73) | 0.199 |

*Rates of change for each factor were compared to pre-treatment.

Abbreviations: IQR = Interquartile Range, ADC = Apparent diffusion coefficient
